# Dynamic Threshold Effect of Directed Technical Change Suppress on Urban Carbon Footprint in China

**DOI:** 10.3390/ijerph19095151

**Published:** 2022-04-23

**Authors:** Xiaojun Lyu, Haiqian Ke

**Affiliations:** 1 Fanli Business School, Nanyang Institute of Technology, Nanyang 473000, China; lxiaojun738240@126.com; 2 Institute of Central China Development, Wuhan University, Wuhan 430072, China

**Keywords:** directed technical change, carbon footprint, dynamic threshold regression

## Abstract

Promoting technical change is an important driving force for promoting the sustainable development of urban economy and ecology; however, the technical change is not always neutral and technical change may has a certain direction. This paper uses the DEA-Malmquist index to measure the directed technical change of 280 cities in China from 2009 to 2019, and uses the DMSP/OLS night light data to characterize the urban economic development level. It uses the dynamic threshold regression model to analyze the impact of directed technical change on urban carbon footprint under different economic development levels. The results show that: (1) during the study period, the carbon footprint of Chinese cities has a positive spatial correlation, and the direction of technical change is towards capital-saving overall. (2) The impact of capital-saving technical change on urban carbon footprint presents a negative double-threshold characteristic in China, and the inhibition of capital-saving technical change on the urban carbon footprint becomes stronger with the increasing economic development level. (3) The inhibitory effect of capital-saving technical change on carbon footprint has regional heterogeneity, and the inhibitory effect of capital-saving technical change on carbon footprint is stronger in eastern China than other regions. (4) Industrial structure, energy structure and innovation efficiency are mediating variables of the inhibitory effect of capital-saving technical change on carbon footprint except for population density.

## 1. Introduction

The climate change brought about by the greenhouse effect will not only have a great impact on the global ecological environment, but will also endanger the production, consumption, lifestyle and living space of human beings [1]. Therefore, in recent years, climate change has been at the top of the global environmental issues and has attracted more and more attention, making it a consensus of the international community to deal with global climate change. Since 2015, at least 190 countries and regions have signed the Paris Agreement, which emphasizes the need to strengthen the global response to the threat of climate change. The core goal is to limit the global average temperature increase to less than 2 °C by the end of the 21st century, and to strive to limit the temperature increase to within 1.5 °C [2]. As of 2020, a total of 54 countries and regions in the world have achieved carbon peaks, mainly in developed countries. As the largest developing country in the world, China’s demand for natural resources has exceeded a reasonable range with the rapid development of urbanization [3]. The total amount of carbon emissions is huge, accounting for about 28% of the world’s total. China’s energy structure is dominated by coal, and in 2019, coal accounted for 57.7% of total energy consumption [4]. In fact, climate change, environmental pollution, and the sharp reduction in total resources have placed varying degrees of ecological pressure on China’s urbanization development, threatening the sustainable development of the urban economy and the environment [5]. Therefore, it is necessary for us to conduct continuous tracking research on environmental issues such as climate change in China.

Under the background of tightening resource constraints and ecological environment degradation, technical change has become one of the most important sustainable development paths for industrial structure upgrading, competitiveness enhancement and environmental sustainability. However, Hicks argued in his 1932 wage theory that technical change leads to an increase in the marginal output of an element when the ratio of capital to labor remains constant, thus giving rise to the direction of technical change, also known as biased or directed technical change [6]. And so, will directed technical change have an impact on urban carbon emissions? Will this impact change as the level of economic development increases? What is more, China is a country with an uneven distribution of natural resources. Does the impact of directed technical change on urban carbon emissions with different resource endowments have regional differences? In view of this, this paper will reveal the dynamic threshold effect on carbon emissions from the perspective of directed technical change, and provide experience for urban sustainable development in China.

## 2. Literature Review

### 2.1. Carbon Emissions

With the increasingly prominent environmental problems arising from the development of industrial civilization, Malthus (1798) proposed the theory of population that “the main cause of resource scarcity comes from the limit growth of population”, which is also the starting point for people to pay attention to whether the population scale has an impact on the environment [7]. Commoner (1971), an American biologist and ecologist, proposed “technological determinism”, believing that the development of industrial technology was the primary cause of environmental quality deterioration [8]. At the same time, the “Population growth Theory” believed that “compared with sophisticated management technology, oversize population is the deep-seated cause of ecological environment pressure”, emphasizing that population growth is the most important cause of environmental deterioration. Thus, the IPAT model is a widely accepted cognitive framework of the impact of economic and social development on the natural environment, that is, the impact of economic and social development on the natural environment is closely related to the size of the human population, the degree of wealth, and the level of technology [9]. However, since the IPAT model belongs to the identity model, and the explanatory variables have an equal proportional derivation relationship with each other, this cannot explain the influence of a single factor on environmental quality [10]. Therefore, the STIRPAT (Stochastic Impacts by Regression on Population Affluence and Technology) model is formed on the basis of the IPAT model. Especially after obtaining the natural logarithm of both sides, more variables are added to the right side of the equation to enhance the richness of the model [11].

“Carbon footprint” is one of the most commonly used indicators used to measure greenhouse gas emissions, which can quantitatively measure the forest area required to absorb the corresponding carbon emissions in a certain area. By comparing this with the local forest area, the local environmental pressure can be judged intuitively, and then, the regional carbon emissions can be compared and analyzed from a multi-dimensional perspective of time and space. The concept of “carbon footprint” is based on the concept of “ecological footprint” [12], which first became popular in the UK. It represents a collection of greenhouse gas emissions caused by an enterprise, activity, product, or an individual. The main greenhouse gas emissions are generated through transportation, food production and consumption, energy use and various production processes. By combing the relevant literature, scholars have done a lot of research on carbon emission and carbon footprint from the different angle, objects and methods. For example, from the product, household, industry, land use, region, country and other different scales of research; from the perspective of consumption and production, there are also direct carbon emissions and indirect carbon emissions [13,14,15,16,17].

**Figure 1:**
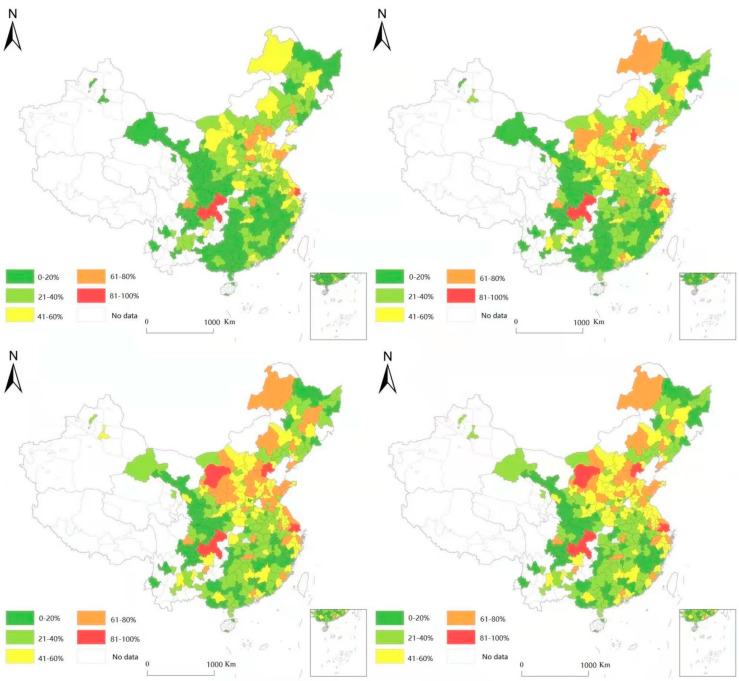
China’s urban carbon footprint in 2009, 2013, 2016, and 2019.

This paper uses ArcGIS to display the spatial distribution map of urban carbon footprint in 2009, 2013, 2016, and 2019 in Figure 1 (the upper left picture is 2009, the upper right picture is 2013, the lower left picture is 2016, and the lower right picture is 2019). There are five categories, sorted numerically from high to low, and each category accounts for 20% of the 280 cities. The closer the color is to red, the larger the carbon footprint of the city, and the closer the color is to green, the smaller the carbon footprint of the city. We can clearly see that the overall level of the urban carbon footprint from 2009 to 2019 is a significantly weakening. Among these, the carbon footprint of the eastern region in 2019 is obviously lower than that in 2009, and it also shows a trend of shifting to the central and western regions during the study period. However, urban carbon footprints in the northeast have not changed much from 2009 to 2019. The possible reason for this is that in recent years, the eastern region has paid more and more attention to the quality of the urban environment, and green and high-quality development has become the main theme of urban development. However, under the national industrial transfer policy, the western region has to take over the transfer of polluting industries from the eastern region, so the carbon footprint of the western region has not decreased significantly from 2009 to 2019 [18,19].

### 2.2. Directed Technical Change

The main endogenous function form is the C-D (Cobb-Douglas) function, which can well describe the phenomenon of balanced economic growth, and the Kaldor stylized fact is consistent with the fact that the ratio of labor and capital income remains stable in the long run. The C-D function, which makes the neutral technical change, has been widely used [20]. However, with the large-scale development of the software machinery and equipment industry in various countries globally, technical change has shown a capital-biased attribute, and the proportion of labor income has also shown a continuous downward trend, which deviates from the traditionally stylized fact that Kaldor’s labor income proportion is stable [21]. Neutral technical change is beginning to be questioned.

Facts have proved that neutral technical change cannot really depict the evolution of technology, and capital-saving technical change may occur in the process of economic transition; that is, technical change may significantly contribute to raising the marginal productivity of capital and increasing the share of capital investment income in national income [22,23]. Moreover, with the decline in the share of labor income and the skill premium in some developed countries [24,25], the direction of technical change theory was effectively extended by Acemoglu (2002, 2006, 2007), whose main contribution is that he extended the direction of technical change to any two factors (*Z*, *L*) and also defined the directed technical change: when the technical change makes the relative marginal output of the factor *Z* increase, we could say that technical change is in favor of the *Z* factor [26,27,28].

### 2.3. The Relationship between Directed Technical Change and Carbon Emissions

The research on the relationship between technical change and carbon emissions is carried out from the following two aspects. First, technical change can significantly affect carbon emissions through energy substitution rate, factor substitution rate and other factors. Bampatsou et al. (2013) used the data envelopment analysis (DEA) method and found that the impact of technical change on carbon emissions comes from two aspects. That is, technical change may increase energy demand while reducing energy consumption. The degree of influence of these two aspects leads to different directions of the impact of technical change on carbon emissions [29]. Schiemann et al. (2020) found that economic growth brought about by technical change will promote carbon emissions, but if technical change brings clean technology, not just technology that improves production efficiency, technical change will effectively reduce carbon emissions [30]. Hui et al. (2020) also found that the reduction of carbon emissions brought about by technical change through energy efficiency improvements cannot yet offset the increase in carbon emissions brought about by its promotion of economic growth [31]. It is worth noting that the research of Gu et al. (2020) shows that the technical change of the previous period can significantly reduce the carbon emissions of the current period, indicating that there is a certain time lag in carbon emissions [32]. Secondly, from the perspective of the impact of directed technical change on carbon emissions, the earlier analysis on the economic effects of environmental policies was mainly carried out under the condition of exogenous technology, ignoring the endogenous problem of environmental technology. Recently, scholars have begun to internalize the directed technical change and introduce it into the climate change model. Smulders and Maria (2012) and Ploeg and Rezai (2016) found that if directed technical change is ignored, the welfare cost of optimal carbon tax will be exaggerated [33,34]. This implies that directed technical change has some impact on carbon emissions. Furuya et al. (2015) found that the direction of technical change in Japan has an important impact on carbon emissions [35]. Kronenberg (2010) verified that directed technical change has a positive effect on China’s industrial energy conservation and emission reduction [36].

In addition to the relationship between directed technical change and carbon emissions, it also needs to be considered that the impact of directed technical change on urban carbon emissions in eastern China may be different to other regions due to the higher level of economic development [37]. For example, Li and Wang (2019) found that the inhibitory effect of technical change on China’s pollutant emissions is positively affected by the level of regional economic development; that is, when the level of economic development is high, the financial support required for the development and application of technical change is more effective, which is conducive to vigorously developing, promoting and utilizing clean energy, thereby reducing carbon emissions and suppressing pollutant emissions [38]. Simultaneously, with the improvement of the level of economic development, the social awareness of environmental protection has been relatively enhanced, and people’s consumption preferences have gradually shifted from the price of the final product to the environmental protection and energy saving of the product production process, which alleviates the growth of total carbon emissions to a certain extent [39]. It can be seen that due to the differences in the level of regional economic development, directed technical change may have varying degrees of impacts on carbon emissions.

Additionally, there are also significant regional differences in directed technical change in China. Due to the excellent technology research and development environment in the eastern region of China, the investment amount of scientific and technological resources and the technology market turnover have shown rapid growth year on year [40]. The technical level has been continuously improved, and the degree of openness is much higher than the national average level, the market economy has a high degree of freedom, the industrial structure has been continuously optimized and upgraded, and the public’s appeal for improving the ecological environment is relatively higher. The DEA method is used to measure the directed technical change of 30 provinces in mainland China, and it was found that the directed technical change in the eastern region was significantly higher than that in the central, western and northeastern regions [41,42]. Therefore, in addition to different economic development stages, directed technical change may have different impacts on carbon footprints, and at the same time, it may have a heterogeneous impact on carbon footprints in different regions, and existing research lacks attention on this issue. Therefore, this paper aims to explore whether there is also a threshold effect on the impact of the directed technical changes on carbon footprint under different economic development levels. Moreover, does the threshold effect change in different regions?

## 3. Models and Data

### 3.1. Models

#### 3.1.1. STIRPAT Model

This paper selects and combines the STIRPAT model as the basic theoretical framework for studying the quality of the ecological environment [10,11]. However, the panel data form of the STIRPAT model is originally from the IPAT model. The IPAT model is as shown in Formula (1):

(1) $$I_{it}=aP_{it}^{b}A_{it}^{c}T_{it}^{d}e\;\mathrm{for}\;\mathrm{city}\;i\;\mathrm{and}\;\mathrm{time}\;$$

where *I*, *P*, *A* and *T* represent environmental impact, population size, per capita wealth and technical level, respectively, and *e* is the error term. However, the IPAT model belongs to the identity model, and the explanatory variables have an equal proportional derivation relationship with each other which cannot explain the influence of a single factor on environmental quality. Therefore, The STIRPAT model is formed on the basis of the IPAT model [40]. Especially after obtaining the natural logarithm of both sides, more variables are added to the right side of the equation to enhance the richness of the model.

(2) $${lncf}_{it}=\alpha_{0}+\alpha_{1}{lnpop}_{it}+\alpha_{2}{lnpop}_{\mathrm{it}}+\alpha_{3}{lndtc}_{it}++\alpha_{4}X_{it}+\varepsilon_{it}$$

Among them, due to the availability of data, *i* is the cross-sectional unit of 280 prefecture-level cities in China, and *t* is the year; the population is *P*, the per capita wealth *A* and the technology level *T* are represented by the population number (*lnpop_it_*), per capita GDP (*lngdp_it_*), and the core explanatory variable is directed technical change (*lndtc_it_*) respectively; *lncf_it_* is the carbon footprint of the explained variable; *X* is a set of control variables; *α*_0_–*α*_3_, *α*_4_ are parameters to be estimated; *ε* is the random disturbance term.

#### 3.1.2. Dynamic Threshold Regression Model

Because the strict assumptions of Hansen’s (1999) static panel threshold model are difficult to meet in the process of practical application, multicollinearity, significant bias and endogeneity among the variables are extremely likely to occur [43]. In order to solve some of the defects of the static threshold model, Kremer (2013) proposed a dynamic threshold model that included the lag term of the explanatory variable, which solved the endogeneity and lag problems of variables to the greatest extent [44,45]. Therefore, in the following formula, it is only assumed that there is a threshold, but in order to make the analysis more accurate, this chapter also sets a double threshold model and a triple threshold model:

(3) $$\begin{array}{l} {lncf}_{it}=\alpha X_{it}+\beta_{1}{lndtc}_{it}\times I(T_{it}\leq \delta_{1})+\beta_{2}{lndtc}_{it}\times I(\delta_{1}<T_{it}\leq \delta_{2})\\ +\beta_{3}{lndtc}_{it}\times I(T_{it}>\delta_{2})+\beta_{4}{lncf}_{it-1}+\beta_{5}{lncf}_{it-2}+C+\varepsilon_{it} \end{array}$$

(4) $$\begin{array}{l} {lncf}_{it}=\alpha X_{it}+\beta_{1}{lndtc}_{it}\times I(T_{it}\leq \delta_{1})+\beta_{2}{lndtc}_{it}\times I(\delta_{1}<T_{it}\leq \delta_{2})\\ +\beta_{3}{lndtc}_{it}\times I(\delta_{2}<T_{it}\leq \delta_{3})+\beta_{4}{lndtc}_{it}\times I(T_{it}>\delta_{3})+\beta_{5}{lncf}_{it-1}+\beta_{6}{\mathrm{lncf}}_{it-2}+C+\varepsilon_{it} \end{array}$$

### 3.2. Data

#### 3.2.1. Variables

① Explained variable: carbon footprint

According to report from Global Footprint Network (GFN) in 2007, “carbon footprint is a part of the ecological footprint, which can be regarded as equivalent to the ecological footprint of fossil energy” [46]. That is to say, the carbon footprint is the geographical area required to absorb the corresponding carbon emissions in a specific area. The larger the carbon footprint, the higher the impact on regional warming. The formula is as follows:

(5) $${lncf}_{it}=\sum {cf}_{i}=\sum C_{i}/F_{i}$$

In Formula (1), *lncf_it_* is the urban carbon footprint; *cf_i_* is the energy utilization carbon footprint of the *i*-th energy; *C_i_* is the per capita carbon emissions of the *i*th energy; and *F_i_* is the land conversion coefficient of the *i*th energy, that is, the actual consumption of various energy sources is converted into carbon emissions. Then, the carbon footprint of various energy use is calculated by the ratio of carbon emissions to land area conversion coefficients [47]. The land area conversion factor (*F_i_*) is 6.49 t/hm^2^ (measured by the amount of CO_2_ absorbed by forest land) [48].

Then, we calculated CO_2_ emissions for 280 cities in China according to the type of fuel consumption. Among them, the types of urban energy consumption mainly include raw coal, coke, crude oil, gasoline, diesel oil, fuel oil, natural gas, heat, electricity. The data of fuel consumption are generally measured and accounted for in mass or volume units. Since the carbon content of fuel is usually closely related to the energy content, it is reasonable to convert the fuel consumption value into calorific value units. Based on the 2006 IPCC (Intergovernmental Panel on Climate Change), the calculation method of the carbon emission coefficient of various energy sources shown in the National Greenhouse Gas Emission Inventory Guide [49], and the calculation method of the relevant CO_2_ emission is as follows:

(6) $$C_{i}=\sum \ln ec_{ij}\times r_{j}$$

In the formula, *r_j_* is the carbon emission coefficient of the *j_th_* energy, and the carbon emission coefficients of various energy sources are shown in the Table 1.

② Core explanatory variables: directed technical change

Fare et al. (1997) decomposed the Malmquist index into the following parts: technical efficiency change index (FFCH) and technology change index (TECH) [48]. It also decomposes the technology change index into the technology scale change (MATECH), the output-biased technology change (OBTECH) and the input-biased technology change (IBTECH) index. As IBTECH can effectively judge the direction of technical progress after combining the changes in the proportion of element input combination between two adjacent periods. Therefore, this article uses the IBTECH index to measure the directed technical progress [50,51,52], and the calculation method is as follows:

Let *x_t_* = (*x*_1*t*_, ……, *x_Nt_*) be the vector of factor input in period *t*, *yt* = (*y*_1*t*_, ……, *y_Nt_*) be the vector of output in period *t*. The Shephard input distance function in period *t* can be defined as:

(7) $$D_{i}^{t}(y,x)=max\{\lambda :\frac{x}{\lambda }\in L^{t}(y)\}$$

Among them, *L_t_*(*y*) represents the possible investment combination required during *t* period. With constant returns to scale, the Malmquist index (*MI*) used to measure total factor productivity is:

(8) $$MI=\sqrt{\frac{D_{0}^{t+1}(y^{t},x^{t})}{D_{0}^{t+1}(y^{t+1},x^{t+1})}\times \frac{D_{0}^{t}(y^{t},x^{t})}{D_{0}^{t}(y^{t+1},x^{t+1})}}$$

*MI* can be broken down into *FFCH* and *MATECH*, *OBTECH* and *IBTECH*:

(9) $$MATECH=\frac{D_{0}^{t+1}(y^{t},x^{t})}{D_{0}^{t}(y^{t},x^{t})}$$

(10) $$OBTECH=\sqrt{\frac{D_{0}^{t+1}(y^{t+1},x^{t+1})}{D_{0}^{t}(y^{t+1},x^{t+1})}/\frac{D_{0}^{t+1}(y^{t+1},x^{t})}{D_{0}^{t}(y^{t+1},x^{t})}}$$

(11) $$IBTECH=\sqrt{\frac{D_{0}^{t+1}(y^{t+1},x^{t})}{D_{0}^{t}(y^{t+1},x^{t})}/\frac{D_{0}^{t+1}(y^{t},x^{t})}{D_{0}^{t}(y^{t},x^{t})}}$$

When *x*2*_t_*_+1_/*x*1*_t_*_+1_ < *x*2*_t_*/*x*1*_t_* and *IBTECH* are both less than 1, the technical change is *x*1*_-saving_*, and the *IBTECH* becomes smaller, the higher the degree of technical change directed towards *x*1*_-saving_*. When *x*2*_t_*_+1_/*x*1*_t_*_+1_ *> x*2*_t_*/*x*1*_t_*, the situation is the opposite. When *x*2*_t_*_+1_/*x*1*_t_*_+1_ = *x*2*_t_*/*x*1*_t_*, technical change is neutral. In this study, *x*1 is the labor input, and *x*2 is the capital input. In order to unify the two situations, this article sums up the opposite of the *IBTECH* value in *x*2*_t_*_+1_/*x*1*_t_*_+1_ > *x*2*_t_*/*x*1*_t_* and two. The *IBTECH* value under *x*2*_t_*_+1_/*x*1*_t_*_+1_ < *x*2*_t_*/*x*1*_t_* remains unchanged to obtain the quantitative index of labor-saving technical change. Therefore, a large value of this index indicates that the directed technical change is more capital-saving.

③ Mediating variables

This paper selects the following four variables to characterize population aggregation, industrial structure, energy structure and innovation efficiency, respectively. Specifically, we use population density to characterize the effect of population agglomeration; the ratio of the secondary industry to GDP to characterize the industrial structure; the ratio of coal consumption to energy consumption to measure the energy structure; and the ratio of input and output of innovation resources to characterize innovation efficiency. Based on the related literature, it is expected that the population agglomeration effect and the improvement of innovation efficiency will reduce the carbon footprint. It is expected that the ratio of the secondary industry to GDP and the ratio of coal to total energy will both contribute to the carbon footprint [53,54].

④ Threshold variable

DMSP/OLS data is currently one of the most widely used nighttime light data, and it has been used in studies on population estimation, electricity consumption estimation, and urban expansion monitoring. Therefore, this paper selects the nighttime light data of DMSP/OLS to characterize the economic development level of 280 cities in China (*lnnl_it_*). Because stable light data can be used not only to test real economic growth, but also to measure economic activities such as economic agglomeration, urbanization, population mobility, and energy consumption [55]. At the same time, stable light data objectively reflects regional differences in the production and living conditions of human society [56].

⑤ Control variables

Based on the STIRPAT model (Dietz and Rosa 1994) [11], this paper selects the following five control variables from the economic, social, and environmental perspectives: the proportion of tertiary industry (*lnthird_it_*), foreign direct investment (*lnfdi_it_*), total road passenger transport (*lntrans_it_*), the number of full-time college teachers (*lntechs_it_*), and the scale of pollution control investment (*lnpollution_it_*) [57,58]. Among them, population, GDP and total road passenger transport are expected to have a positive effect on carbon footprint. However, the proportion of tertiary industry, foreign direct investment, the number of full-time college teachers and the scale of pollution control investment are expected to have a suppressing effect on carbon footprint.

#### 3.2.2. Data Source

Considering the availability and representatives of data, this paper takes 280 cities in China as the research object, and obtains the basic data for measuring urban energy consumption and carbon footprint from the relevant yearbooks from 2009 to 2019; “China Statistical Yearbook”, “China Environmental Statistical Yearbook, China Forestry Statistical Yearbook, China Social Statistical Yearbook, Compilation of Foreign Resources, Energy and Environment Statistical Data, China Rural Statistical Yearbook”, etc. The capital stock of urban-oriented technological progress is estimated by the perpetual inventory method, with 2000 as the base period, of which the total employment and real GDP are derived from the 2010–2020 China Urban Statistical Yearbook. This paper uses 2009–2019 DMSP/OLS data to obtain nighttime light data in 283 cities in China, and uses the method of Xu et al. (2020) to calibrate the problem of inconsistent data sources around 2013 [59]. This paper obtains the basic data for calculating the DEA-malmquist index from the China Urban Statistical Yearbook. The input variables are labor and capital. The labor is estimated by the total number of employees in the region, and the capital is estimated by the capital stock. The output variable is the real GDP of the region.

## 4. Results Analysis

### 4.1. Regional Difference Analysis of Directed Technical Change on Carbon Footprint

The 280 cities are classified according to the regions of China’s four major plates, including 100 cities in eastern China, 79 cities in central China, 68 cities in western China and 33 cities in northeastern China. According to Formulas (3) and (4), the Hausman significance test was performed on the panel data, and the results showed that the null hypothesis was rejected, so the fixed-effects model was selected for analysis. Based on the fixed effect model, the carbon footprint is used as the explained variable; the night light data (replacing the level of economic development) is used as the threshold variable. This paper measures the impact of urban technological change on the carbon footprint of the whole country, eastern, central, western and northeastern regions under different economic development levels.

All regions have passed the single-threshold test in Table 2, but there are obvious differences in their significance. The single-threshold test of the eastern and northeastern regions is only at the 10% level, and the significance of the central and western regions is at the 5% level. However, all regions except the northeast region passed the 1% significance level for the double-threshold test; the triple-threshold effect in the west and northeast regions was not significant. Therefore, under the premise that night light data are used as the threshold variable, the F value of the double threshold of capital-saving technical change on carbon footprint is relatively large. Therefore, this paper adopts the double-threshold test for the impact of capital-enhanced technological progress on carbon footprint.

It can be seen from Table 3 and Table 4 that the first line and second line show that there is a positive spatial relationship between urban carbon footprint of China, and this is also consistent with the conclusions from some scholars [60,61,62]. When the first and second lag periods of urban carbon footprint are used as the explained variables, for the whole country, when the urban night light data is below the first threshold of 6864.52, the elasticity coefficient of capital-saving technical change on carbon footprint is −0.2077, and it is significant at the 1% level. When the urban night light data are between the two thresholds, the elasticity coefficient of capital-saving technical change to carbon footprint is −0.3003. When the night light data exceed the second threshold of 8136.44, the elasticity coefficient of capital-saving technical change to carbon footprint is −0.3428. Therefore, under the dynamic threshold model, the improvement of China’s overall urban capital-saving technical change significantly inhibits the growth of carbon footprint, and at the same time, the higher the city’s night light data, the stronger the inhibition effect.

Similarly, the inhibitory effect of capital-saving technical change on the carbon footprint in the eastern, central and western regions is consistent with that of the country as a whole. That is, the improvement of capital-saving technical change has an increasingly strong inhibitory effect on the growth of carbon footprint in the eastern, central and western regions. Among them, for the western region, when the night light data exceed the first threshold of 6780.19 and the second threshold of 8469.38, the effect of increasing capital-saving technical change on curbing total carbon footprint is becoming more and more obvious, with the coefficient changing from −0.0038 to −1.0003 (all at the 1% significant level). It is worth mentioning that for the northeast region, only after the night light data crosses the second threshold of 10,705.68 does capital-saving technical change exhibit a dampening effect on the carbon footprint with a coefficient of −1.0216, which is significant at the 5% level. Therefore, under the dynamic threshold model, we control for the endogeneity of the explained variables to a certain extent, the impact of capital-saving technical change on urban carbon footprint still has significant stage and regional differences.

Regarding the control variables, except for the population in the western region, which showed a significant promoting effect on the urban carbon footprint in all models, GDP shows a significant contribution to the city’s carbon footprint. The level of foreign direct investment, the ratio of tertiary industry to GDP, and the ratio of pollution input scale to GDP have significant inhibitory effects on carbon footprint in all models; the annual passenger volume of highways has no significant impact on the total urban carbon footprint, so there is no regional difference. The improvement of education level represented by the number of college teachers only shows a restraining effect on carbon footprint in the eastern region, which shows that the public’s environmental protection awareness has a certain relationship with the urban social and economic development level [63].

### 4.2. Mediation Effect Regression Results

It can be seen from Table 5 that the coefficients of *lndtc_it_* in model (2) are −1.9421, with 1% significant level, and the Sobel value is 0.8703, which is less than 0.97. This means that the increase in the level of capital-saving technical change does not inhibit or promote urban carbon footprint by changing population density or population aggregation; that is, population density as a mediating variable does not play a significant mediating effect. This may be because there is no significant direct association between capital-saving technical change and population density, so the mediating effect does not exist. The coefficients of *lndtc_it_* in model (5) are −0.4709, respectively, with 1% significant level, and the Sobel value is greater than 0.97. This means that the improvement of the level of capital-saving technical change can inhibit the urban carbon footprint by changing the industrial structure. That is, the industrial structure as an intermediary variable has played a significant mediating effect. However, in models (4) and (6), the coefficient of *lndtc_it_* is significant at different levels, indicating that this is not a full mediating effect, but a partial mediating effect.

The coefficients of *lndtc_it_* in model (2) and (5) are −1.3706 and −2.0505, respectively, and both are significant at the 1% level, and the Sobel value is greater than 0.97. This means that the improvement of the level of capital-saving technical change can inhibit the urban carbon footprint by changing the energy structure; that is, the energy structure as an intermediary variable has played a significant mediating effect. However, in model (1) and (3), the coefficient of *lndtc_it_* is significant at the 1% level, indicating that this is not a complete mediating effect, but a partial mediating effect. It can be seen from Table 6 that the coefficients of *lndtc_it_* in model (5) are −2.0505, and with 1% significant level, and the Sobel value is greater than 0.97. This means that the increase in the level of capital-saving technical change has an inhibitory effect on urban carbon footprint by changing innovation efficiency. That is, innovation efficiency as an intermediary variable has played a significant mediating effect. However, in models (4) and (6), the coefficient of *lndtc_it_* is significant at the 1% level, indicating that this is not a full mediating effect, but a partial mediating effect.

## 5. Conclusions

This paper investigates the relationship between directed technical change (capital-saving) and carbon footprint under a dynamic threshold effect model, and then discusses whether the relationship will change in different regions across China. Then, we use a mediation effect model to partially test the impact mechanism of capital-saving technological progress on carbon footprint: (1) the direction of technical change is towards capital-saving among Chinese cities. The carbon footprint of Chinese cities has a positive spatial correlation (2) the inhibition of capital-saving technical change on urban carbon footprint becomes stronger with the increase of economic development level. (3) The inhibitory effect of capital-saving technical change on carbon footprint has regional heterogeneity, and the inhibitory effect of capital-saving technical change on carbon footprint is stronger in eastern China than other regions. (4) The mediating variables of the inhibitory effect of capital-saving technological change on carbon footprint include industrial structure, energy structure and innovation efficiency.

Therefore, first of all, while improving the level of technological progress, the Chinese government should also pay attention to the direction of technological progress, so as to better strengthen the inhibition of carbon footprint by adjusting the industrial structure and energy structure. Secondly, it is important to formulate differentiated strategies according to the resource endowments of different cities, so that cities can achieve sustainable development in terms of economic development and environmental protection. Thirdly, the central city should play the leading role of its growth pole to a greater extent, drive the surrounding cities to improve the level of technological progress, and strengthen their role in carbon footprint. More importantly, although this paper is innovative in its research perspective, most of the literature only pays attention to the impact of the level of technological progress on carbon footprint, ignoring the importance of the direction of technological progress. However, in this paper, remote sensing data can be used to be more detailed and accurate. The research scope can also be broader. For example, advanced technology must be used in the process of emission reduction, and the extensive use of technology will consume a lot of energy. For example, electricity. Therefore, the direction of our future research can start from distinguishing the relationship between energy saving and emission reduction.

## Figures and Tables

**Table 1:** Carbon emission coefficients of various energy sources.

Energy Types	Raw Coal	Coke	Crude Oil	Gasoline	Diesel Oil	Fuel Oil	Natural Gas	Heat	Electricity
coefficients	0.4861	0.7482	0.8206	0.8071	0.8453	0.8657	5.3903	0.0279	0.1623

**Table 2:** Threshold effect test.

Regions	National	Eastern	Central	Western	Northeastern
single threshold test	41.565 ***	19.203 *	26.187 **	19.054 **	22.019 *
	(5.29)	(1.78)	(2.20)	(2.01)	(1.70)
double threshold test	28.005 ***	17.146 ***	22.436 ***	20.183 ***	27.043 **
	(3.09)	(4.17)	(6.88)	(5.90)	(2.09)
triple threshold test	11.001 *	9.076 **	12.261 ***	8.238	0.000
	(1.77)	(1.99)	(6.01)	(0.47)	(0.12)

Note: ***, ** and * indicate significance at the 1%, 5% and 10% levels, respectively.

**Table 3:** Threshold estimate.

	Single-Threshold Estimate (*δ*_1_)	95% Confidence Interval	Double-Threshold Estimate (*δ*_2_)	95% Confidence Interval
Entire country	6864.52	(5903.09, 7215.83)	8136.44	(7965.84, 9019.78)
East	5824.17	(4978.69, 6070.11)	7211.86	(6553.07, 7422.84)
Central	7802.36	(7255.39, 8104.61)	8427.30	(7909.68, 8577.93)
West	6780.19	(6407.63, 6978.26)	8469.38	(7719.36, 9066.78)
Northeast	9245.32	(8905.83, 9763.15)	10,705.68	(9758.89, 11,003.25)

**Table 4:** Dynamic threshold regression results of capital-saving technical change on carbon footprint in different regions.

Variables	Model (1)	Model (2)	Model (3)	Model (4)	Model (5)
	Entire Country	East	Central	West	Northeast
*lnec_it-_*_1_	2.0725 ***	1.2073 ***	1.0413 ***	1.6073 ***	1.0266 ***
	(4.13)	(3.06)	(5.88)	(4.01)	(6.06)
*lnec_it-_*_2_	1.8547 ***	0.9877 ***	0.8765 ***	0.9463 ***	0.9029 ***
	(3.24)	(4.17)	(5.78)	(4.09)	(3.62)
*lndtc* (*T_it_* < *δ*_1_)	−0.2077 ***	−1.0208 ***	−0.0097 ***	−0.0038 **	−0.7021
	(−5.73)	(−4.17)	(−3.08)	(−2.10)	(0.88)
*lndtc* (*δ*_1_ ≤ *T_it_* < *δ*_2_)	−0.3003 ***	−1.0302 ***	−0.1015 ***	−0.0705 ***	−0.9006
	(−3.01)	(−4.47)	(−5.13)	(−3.68)	(1.20)
*lndtc* (*T_it_* ≥ *δ*_2_)	−0.3428 ***	−1.3063 ***	−0.2705 ***	−1.0003 ***	−1.0216 **
	(−3.17)	(−5.44)	(−3.35)	(−5.08)	(−1.98)
*lnpop* *_it_*	0.9037 ***	0.8025 ***	1.0004 ***	−0.0713 ***	0.5946 ***
	(3.79)	(−3.29)	(6.09)	(−5.17)	(−4.16)
*lngdp* *_it_*	1.0005 ***	0.9801 ***	0.7975 ***	0.8009 ***	0.0429 ***
	(3.91)	(3.83)	(3.46)	(4.08)	(5.91)
*lnfdi* *_it_*	−0.9358 ***	−1.0133 ***	−0.2046 ***	−0.7085 ***	−0.1129 ***
	(−4.55)	(−5.06)	(−5.97)	(−4.83)	(−6.04)
*lnthird* *_it_*	−0.9031 ***	−0.6708 ***	−0.4079 ***	−0.3397 ***	−0.4289 ***
	(−4.00)	(−3.46)	(−4.82)	(−7.03)	(−5.62)
*lntrans* *_it_*	0.0740	0.0526	0.0899	0.0645	0.1012
	(0.45)	(1.02)	(1.23)	(0.97)	(1.38)
*lntechs* *_it_*	−1.0802	−0.9153 ***	−1.0246	−0.9011	−1.2038
	(−0.94)	(−1.02)	(−0.68)	(−0.93)	(−1.01)
*lnpollution* *_it_*	−1.2463 ***	−1.0589 ***	−0.9976 ***	−1.0205 **	−1.6173 ***
	(−4.07)	(−3.28)	(−4.03)	(−2.39)	(−3.04)
C	6.533 ***	5.087 ***	3.014 ***	4.006 ***	3.498 ***
	(7.33)	(5.10)	(3.47)	(3.96)	(5.08)

Note: *** and ** indicate significance at the 1% and 5% levels, respectively.

**Table 5:** Mediation effect regression results of population density and industrial structure.

Variables	Model (1) *lnec_it_*	Model (2) *lndens_it_*	Model (3) *lnec_it_*	Model (4) *lnec_it_*	Model (5) *lnins_it_*	Model (6) *lnec_it_*
*lndens_it_*			−0.0987 **			−0.9045 ***
			(−2.20)			(−3.67)
*lndtc_it_*	−0.0804 ***	−1.9421 ***	−0.9478 ***	−0.2766 ***	−0.4709 ***	−0.6570 **
	(−4.05)	(−3.00)	(−2.71)	(−4.88)	(−3.70)	−2.5
*lnpop_it_*	0.0608 ***	0.0931 ***	0.0833 **	0.7302 ***	0.4331 **	0.5062 ***
	−5.47	−3.99	−2.39	−4.07	−2.22	−5.78
*lngdp_it_*	0.7576 ***	0.0922 **	1.0273 ***	0.0994 ***	0.0871 ***	0.9204 ***
	−4.67	−2.12	−4.08	−3.27	−3.1	−4.45
*lnfdi_it_*	−0.9760 ***	−0.6834 **	−0.0901 ***	−1.0742 **	−0.9776 **	−0.3802 ***
	(−2.99)	(−2.07)	(−3.88)	(−2.62)	(−2.47)	(−5.06)
*lnthird_it_*	−0.4698 ***	−0.0926 **	−0.6609 ***	−0.0076 ***	−0.0328 *	−0.1059 ***
	(−3.93)	(−1.98)	(−4.04)	(−3.97)	−1.84	(−4.75)
*lntrans_it_*	0.0411 ***	−0.0289 ***	0.0738 ***	0.3776 ***	0.2588 ***	0.0901 ***
	(−4.83)	(−5.07)	(−4.56)	(4.19)	−5.32	−3.66
*lntechs_it_*	−0.0095	−0.0702	−0.0548	−0.1023	−0.3864	−0.8991
	(−0.01)	(−0.49)	(−1.32)	(−0.99)	(−1.37)	(−0.71)
*lnpollution_it_*	−1.2205 ***	−0.1988 ***	−0.4759 ***	−0.1209 ***	−0.3765 ***	−0.8201 ***
	(−4.30)	(−3.41)	(−3.26)	(−4.48)	(−3.70)	(−3.26)
Time fixed effect	Control	Control	Control	Control	Control	Control
Individual fixed effects	Control	Control	Control	Control	Control	Control
Constant	0.1920 ***	−0.9928 ***	0.8330 ***	3.8029 ***	−4.7280 ***	3.6004 ***
	−3.93	(−3.30)	−4.99	−3.43	(−4.65)	−6.03
R^2^	0.758	0.7869	0.7761	0.7361	0.7822	0.7553
Sobel	|Z| = 0.8703 **			|Z| = 2.6935 **		
Mediating effect	No mediating effect			Partial mediating effect		

Note: ***, ** and * indicate significance at the 1%, 5% and 10% levels, respectively.

**Table 6:** Mediation effect regression results of energy structure and innovation efficiency.

Variables	Model (1) *lnec_it_*	Model (2) *lnens_it_*	Model (3) *lnec_it_*	Model (4) *lnec_it_*	Model (5) *lnie_it_*	Model (6) *lnec_it_*
*lndens_it_*			−0.9877 ***			−0.9832 ***
			(−3.70)			(−6.26)
*lndtc_it_*	−0.1921 ***	−1.3706 ***	−0.4559 **	−0.1937 ***	−2.0505 ***	−1.0841 **
	(−5.17)	(−4.65)	−2.33	(−4.51)	(−3.89)	−2.45
*lnpop_it_*	0.7325 ***	0.6822 **	0.0905 ***	0.0681 ***	0.4073 **	0.0943 ***
	−6.1	−2.24	−4.15	−4.28	−2.17	−4.05
*lngdp_it_*	0.0916 ***	0.8807 **	0.1065 ***	0.9925 ***	0.9028 **	1.0085 ***
	−4.09	−2	−3.9	−3.85	−2.19	−5.68
*lnfdi_it_*	−0.1050 **	−0.3629 **	−0.7609 ***	−0.9630 **	−0.8240 **	−0.9064 ***
	(−2.18)	(−2.46)	(−4.57)	(−2.21)	(−2.36)	(−3.69)
*lnthird_it_*	−0.9903 ***	−0.6304 *	−0.4937 ***	−0.6977 ***	−1.0025 **	−0.7793 ***
	(−4.03)	−1.86	(−3.69)	−4.08	−5.78	−4.32
*lntrans_it_*	0.9607 ***	0.8219 ***	0.6503 ***	0.9118 ***	0.9376 ***	0.8762 ***
	−4.66	−7.9	−4.89	−3.95	−4.26	−5.07
*lntechs_it_*	−1.3227	−0.8746	−0.9972	−0.9950	−0.0617	−1.0815
	(−0.98)	(−1.21)	(−0.79)	(−0.87)	(−1.06)	−0.69
*lnpollution_it_*	−0.0736 ***	−0.1028 ***	−0.0977 ***	−0.5876 ***	−0.4210 ***	−0.3599 ***
	(−3.28)	(−5.72)	(−5.68)	(−4.15)	(−3.09)	(−4.77)
Time fixed effect	Control	Control	Control	Control	Control	Control
Individual fixed effects	Control	Control	Control	Control	Control	Control
Constant	2.8240 ***	−5.9070 ***	3.6094 ***	1.0705 ***	−1.6368 ***	3.9784 ***
	−6.73	(−4.22)	−3.94	−5.21	(−4.16)	−3.77
R^2^	0.7582	0.7981	0.7802	0.7258	0.7387	0.7972
Sobel	|Z| = 2.0065 **			|Z| = 1.9896 **		
Mediating effect	Partial mediating effect			Partial mediating effect		

Note: the data in the table are the F statistics corresponding to the threshold test, ***, ** and * indicate significance at the 1%, 5% and 10% levels, respectively, and the P statistics are in brackets.

## Data Availability

Due to the confidentiality and privacy of the data, they will only be provided upon reasonable request.
